# Approximate Sensory Data Collection: A Survey

**DOI:** 10.3390/s17030564

**Published:** 2017-03-10

**Authors:** Siyao Cheng, Zhipeng Cai, Jianzhong Li

**Affiliations:** 1 School of Computer Science and Technology, Harbin Institute of Technology, Harbin 150001, China; lijzh@hit.edu.cn; 2 Department of Computer Science, Georgia State University, Atlanta, GA 30303, USA; zcai@gsu.edu

**Keywords:** approximate computation, sensory data collection, internet of things, wireless sensor networks

## Abstract

With the rapid development of the Internet of Things (IoTs), wireless sensor networks (WSNs) and related techniques, the amount of sensory data manifests an explosive growth. In some applications of IoTs and WSNs, the size of sensory data has already exceeded several petabytes annually, which brings too many troubles and challenges for the data collection, which is a primary operation in IoTs and WSNs. Since the exact data collection is not affordable for many WSN and IoT systems due to the limitations on bandwidth and energy, many approximate data collection algorithms have been proposed in the last decade. This survey reviews the state of the art of approximate data collection algorithms. We classify them into three categories: the model-based ones, the compressive sensing based ones, and the query-driven ones. For each category of algorithms, the advantages and disadvantages are elaborated, some challenges and unsolved problems are pointed out, and the research prospects are forecasted.

## 1. Introduction

From the physical world to the cyber world, the Internet of Things (IoTs) and wireless sensor networks (WSNs) have become necessary connections between them, and make it possible for people to observe the physical world at a low cost. Among all the components of IoT systems and WSNs, the sensory data, as the information carriers, are quite important for both IoT systems and WSNs.

**Figure 1:**
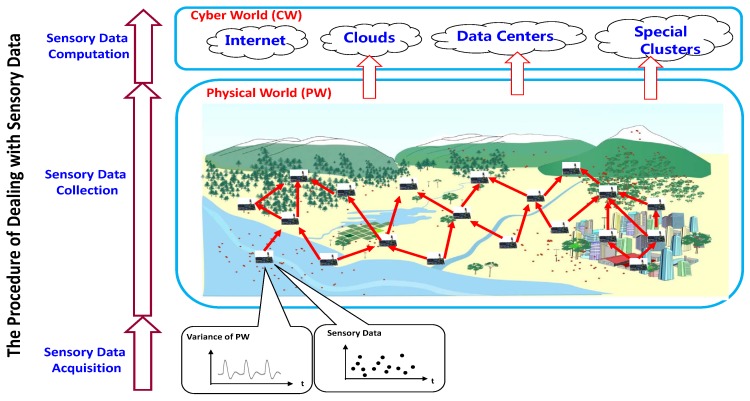
The procedure of dealing with sensory data in IoT Systems.

Usually, the procedure of dealing with sensory data in IoT systems and WSNs can be divided into three phases as shown in Figure 1.

(1) **Sensory Data Acquisition.** In the first phase, the sensing equipment samples the sensory data from the monitoring physical world, which can be seemed as a discrete process for a continuously varying physical world.
(2) **Sensory Data Collection.** In the second phase, the raw sensor data sampled by each sensor node are transmitted into the network toward the sink (sinks), where the sink node is a special node to connect an IoT system or WSN to the cyber world, such as clouds, data centers, etc., and transmit data information between them.
(3) **Sensory Data Computation.** When the sensory data have been already delivered to the cyber world in the second phase, the further computations, e.g., numerical analysis, knowledge discovery, etc., will be carried out according to the applications and users’ requirements.

All of the three phases above are necessary and fundamental for any application of IoT systems and WSNs, and this survey will focus on the second phase. Since the aim of the data collection phase is to transmit the sensory data from the sensors to the sink node, the input of such a phase is the raw sensory values sampled by the sensors in the network, and the output is the sensory dataset received by the sink.

The naive method for sensory data collection is to deliver every raw sensor value sampled by the sensors to the sink node, and we call it the exact sensory data collection methods. Such an exact data collection method guarantees that all the sensory data information are transmitted to the cyber world; however, it also increase the network’s burden with the growth of sensory data.

With the development of the corresponding techniques, including microelectronics, the embedded systems, the wireless communication and sensing techniques, in recent years, the scale of sensory data manifest an explosive growth. According to the annual report of Gartner Inc., (Stamford, CT, USA), which is a famous American research and advisory firm providing information technology related insight for IT and other business leaders, 6.4 billion connected things will be in use worldwide in 2016, up 30 percent from 2015, and will reach 20.8 billion by 2020 [1]. Meanwhile, based on the forecast of Cisco (San Jose, CA, USA), it is estimated that more than 250 things will connect each second by 2020, and it is believed that more than 50 billion things will be connected to the Internet by 2020 [2]. Such a large number of smart “things”, which have sensing abilities, in IoTs will generate a huge amount of sensory data. Furthermore, the volume of worldwide climate data is about 2.5 Petabytes in 2010 estimated by the World Climate Research Program (WCRP), and it is expected to exceed 100 Petabytes in 2020 [3]. The Large Hadron Collider System in Europe deploys 150 million sensors and generates 500 Exabytes sensory data everyday [4]. The taxi monitoring system in Beijing, China produces 48 Petabytes GPS data and 1440 Petabytes other sensory data annually by monitoring 67,000 taxis.

Obviously, such a great amount of sensory data already exceeded the transmission abilities of IoT systems and WSNs; therefore, the exact data collection method is not affordable by many WSN and IoT systems due to the limitations on bandwidth and energy. On the other hand, considering that most sensory data are spatially and temporally correlated since the monitored physical world always continuously varies in space and time [5], the exact sensory data collection is not necessary since a sensory data set are highly redundant due to such strong correlation. Therefore, even if exact data collection is not possible for a WSN or an IoT system, most analysis and computation tasks can still be successfully carried out using approximate data sets that only involve partial data, instead of exact data sets which involve all of the data.

Thanks to the correlation feature of sensory data, many approximate sensory data collection algorithms have been proposed. In this survey, we classify them into three categories, which are the model-based ones, the compressive sensing based ones, and the query-driven ones. This survey summarizes the state of the art of the existing approximate data collection techniques, elaborates their advantages and disadvantages, and points out some challenges and unsolved problems. Finally, we also forecast the research prospects in the future.

## 2. Model-Based Approximate Data Collection Algorithms

One of the earliest pieces of research on approximate data collection algorithms is based on the mathematical models. Usually, the algorithms belonging to this category mainly consist of the following steps.

Firstly, a mathematical model is selected to describe the correlations among sensory data, and the parameters of the chosen mathematical model are learned from the history sensory data.

Secondly, the local prediction models are built according to all parameters obtained in the step above. Meanwhile, all the parameters of the local models are transmitted to the sink for constructing the global prediction model.

Finally, when the local and global models have already been constructed, a newly arrived sensory value does not need to be transmitted if it can be predicted by the local model. Only the sensory values that cannot be predicted by the local model are required to be transmitted to the sink, and the sink node will use the global model to estimate the sensory values that are filtered by the local models.

The main challenges of such approximate data collection methods are how to construct and maintain the local and global models in order to make them be valid all the time. Meanwhile, the main advantage of these methods is that they only transmit a partial amount of sensory data to the sink node since a number of sensory values can be predicted by the models. The detailed information about the algorithms belonging to this category is as follows.

### 2.1. The Algorithms

The initial work in [6] proposed a statistics prediction model based on multivariate Gaussian distribution towards historical sensory data. Sensory data with larger estimation confidence do not need to be transmitted to the sink. This work also considers how to design a query plan to optimize resource consumption. However, it does not consider network dynamics and still requires a large amount of historical data to guarantee performance. Meanwhile, the model cannot detect abnormal events since it relies on historical data.

Another probabilistic model was proposed in [7]. According to the temporal and spatial correlations of sensory data, local and global probabilistic prediction models are respectively built at the sensor nodes and sink. If a node finds that the current sensory value can be estimated by the local model with high probability, it will not send the value to the sink and the sensory value will be estimated according to the global prediction model. An energy efficient *k*-coverage algorithms based on probability driven mechanism is proposed by [8] in wireless sensor networks, besides, the problems of how to schedule the low-energy nodes, how to balance the energy consumption and optimize the network resource was also considered. However, it will lead to the extra energy consumption for maintaining the probabilistic model in the network, meanwhile, such model is also a little ideal and cannot describe the complicate relationship among sensory data accurately.

A spatial-correlation based data collection algorithm was designed in [9]. A network is divided into clusters and sub-clusters periodically such that the sensory data in one sub-cluster are similar and highly spatially correlated. During the data collection process, only a partial amount of data needs to be collected, while other non-transmitted data in the same sub-cluster can be estimated by the Multivariate Gaussian model. However, the model incurs huge energy consumption due to the necessity of frequently updating the sensory data required by the Multivariate Gaussian model. It also seems too strict to assume that sensory data follow the Multivariate Gaussian Distribution.

In [10], similarly, a network is divided into clusters. Instead of Gaussian Distribution, a linear regression model is adopted to describe the temporal correlation of sensory data from a node, and a correlation graph is utilized to depict the spatial correlation of different nodes in a cluster. Only representative nodes of each cluster selected from the minimum dominant set are responsible for reporting data to the sink. Similar to [9], the efficiency and effectiveness of the algorithm depends on the variance of sensory data. If sensory values vary frequently, the models become invalid quickly.

The work in [11] proposed an approximate data collection algorithm in wearable sensor networks utilizing the temporal and spatial correlations among sensory data. The algorithm has two parts. The first part is to determine the transmission order offline, where history data and linear regression are used to analyze the correlations among different sensors. Then, a weighted directed graph is constructed according to the correlations, and the topology order of the weighted directed graph is used to determine the transmission order of different sensors. The second part is to forward sensory data based on the transmission order. Each node overhears the sensory values from the sensors with higher priorities, calculates the difference between its values and the overheard ones, and only reports the difference to the sink. The computation cost for determining transmission order is high and the delay of data collection is unacceptable for large-scale networks; thus, the algorithm is only suitable for small-scale networks.

A flexible framework for mobile data collection is proposed by [12] in health care applications. Such a framework supports the model-based data collection techniques; however, the detailed algorithms were not provided. In [13], a stochastic model, which is based on the conditional probability and priori distribution, is proposed to estimate the vehicle speed trajectories during data collection in vehicle sensor networks. On condition of maximizing the likelihood function, the optimal activity sequence was determined, and then a detailed vehicle speed trajectory would be reconstructed accordingly. Since much trajectory data is estimated instead of retrieving it from the sensor nodes, many energy and transmission costs are saved. Finally, the authors applied this algorithm to a large-scale vehicle dataset and verified the high performance. Such a model only serves for the regular trajectory prediction application, and not applicable for other applications such as abnormal event detection*.etc*. The works in [14,15] also have introduced the Traffic Estimation and Prediction Systems (TrEPS) in VANET, which generally provide the predictive information needed for proactive traffic control and traveler schedule. Such system enhance the performance of the tradition transportation systems, and their prediction models are also useful to reduce amount of sensory data during data collection in VANET, however, they has the similar problem with the one in [13].

An adaptive data collection problem is addressed by [16] in wireless body sensor networks (WBSNs). In traditional WBSNs, each biosensor node collects data and sends them to the coordination in a periodic manner. Therefore, a huge amount of data is collected, which brings a huge burden for transmission and processing. Due to such motivation, the authors proposed a model to reduce the amount of data during data collection on the condition that the integrity of the sensory data is guaranteed. According to their model, each sensory value is scored based on an early warning score system, and the newly coming sensory values won’t be collected if it has the same score as the previous one. Based on such a model, the data fusion method is also proposed using a decision matrix and fuzzy set theory. The algorithm in [16] largely reduced the transmission costs during data collection. However, the proposed model seems a little simple so that such a data collection method is only suitable for emergence detection application.

In [17], the authors surveyed the hierarchical data gathering protocols in WSNs, they have discussed energy and communication overhead for each category of routing protocol with respect to design trade-offs, and also points out the advantages and disadvantages of each technique as well. However, it mainly focused on the exact data gathering protocols and not the approximate ones.

### 2.2. Summary

All of the aforementioned algorithms try to capture the spatial and temporal correlations among sensory data by mathematical models such as Gaussian distribution, linear regression, etc. Based on these mathematical models, many sensory values can be predicted without transmission, so that a large amount of energy will be saved. However, these methods all have the following drawbacks. Firstly, the mathematical models are too ideal to describe the complicated correlations among sensory data accurately. With the wide use of WSNs and IoT systems, the objects monitored by them becomes more and more intricate, and the correlations among sensory data sampled from the monitoring objects are quite complicated as well, so that they may not follow a specific mathematical model, which has colossal impact on the accuracy of the model-based data collection methods. Secondly, they introduce extra in-network communication costs for guaranteeing the correctness of the prediction models and keeping the consistency of the local prediction model and global prediction model. Meanwhile, these algorithms always have a fixed error bound and cannot automatically adjust according to the user-specified precision requirement.

## 3. Compressive Sensing Based Approximate Data Collection Algorithms

Compressed sensing methods have recently become one of the most popular techniques for approximate data collection. Generally, the algorithms belonging to this category regard the sensory data sampled by all the sensors in a given time interval as a matrix with n×m size, where *n* is the size of the network, *m* is the number of sampling times of each sensor in the given time interval, and the algorithms assume all sensors are synchronous in the network and the sensory data matrix is sparse in certain subspaces.

Based on the above assumption, the compressive sensing based approximate data collection algorithms mainly have the following steps.

Firstly, a series of random vectors are generated to form the bases of a subspace.

Secondly, the sensory data is compressed according to the above subspace by projecting the original data matrix into the above subspace.

The main advantages of the algorithms in this category are that the compression rate, i.e., ratio of the dimension of the compressed data matrix to *n*, can be controlled easily according to users’ requirements, and the compression process can be implemented in a distributed manner after the bases are determined. On the other hand, the main challenge of the algorithms is how to generate the appropriate bases. According to the methodology for generating the random bases, the existing works can be classified into the following three groups.

### 3.1. Centralized Algorithms for Generating Compression Bases

This group of algorithms generate the bases of the subspace for compression in a central manner. The common advantage of these algorithms is that they guarantee that the bases are orthogonal with each other so that redundant information can be minimized after compression. The details are as follows.

The work in [18] introduced a method by assuming that the sink has already received partial sensory data. Furthermore, the sampled data are assumed to be compressible and the optimal basis used for compression is known beforehand. In order to collect additional data, the algorithm first randomly samples some data from the network. Then, a vector *p* indicating the set of additional nodes that need to transmit their data is determined centrally according to the received data and the optimal compressing basis. *p* is then broadcasted in the network, and data are transmitted and aggregated from the nodes specified in *p* toward the sink. Obviously, the assumption of obtaining the optimal compressing basis without any knowledge on data distribution is too strong. Even if we can obtain the optimal compressing basis according to historical data, updating the basis incurs a huge amount of energy in WSNs. Furthermore, the delay of data collection is large since *p* needs to be determined iteratively, and a complicated computation needs to be carried out. Therefore, the algorithm is not suitable for large-scale WSNs with frequently varied data.

The works in [19,20] indicate that the *K*-restricted isometry property should be satisfied if we want to recover *N* sensory values from *M* compressed data in polynomial time, where *N* is the total number of sensory values, *K* denotes the sparsity of these values and *M* (M<N) can be determined by *K*. In order to satisfy the above conditions, the work in [21] constructs a matrix to compress sensory data during transmission. A chain network is used as an example and it is proved that [IR] is the matrix guaranteeing the *K*-restricted isometry property, where *I* is the M×M unit matrix and *R* is an M×(N−M) random matrix. This work is also extended to the spanning tree based networks.

Another work considering the *k*-restricted isometry property for compressed sensing [22] intends to recover original sensory data efficiently. The adopted network is an energy harvesting WSN, where the sensors can gain energy from the environment, e.g., wind or solar energy. However, in order to guarantee the *k*-restricted isometry property, the compression size must be determined centrally. Meanwhile, many parameters such as sparsity of data and transmit probabilities must be known by the sink in advance, which is almost impossible for large-scale networks.

The algorithm in [23] uses a function to denote the relationship between sensory data and a sensor node ID, where the base of the function is constructed through a Discrete Cosine Transform. It is assumed that the coefficient of the function is sparse according to the base, such that the compressed sensing technique can be used to compress these coefficients. However, this assumption is too strong for WSNs. Moreover, the accuracy for recovering the original data cannot be guaranteed. Furthermore, the considered topology in this work is not practical for real WSNs.

### 3.2. Distributed Algorithms for Generating Compression Bases

The aforementioned centralized algorithms consume much energy since all the bases need to be broadcasted in the network. This motivates the design of distributed algorithms.

A typical work is proposed in [24], where the matrix for compressing sensory data during collection is constructed in a distributed manner. The main steps are as follows. First, each node generates random vectors according to its ID and the random seed is broadcasted by the sink. Second, for each sensor, a new data vector is obtained by the multiplication of its sensory values and the random vector. Finally, the sink computes the random vectors based on the random seed and sensors’ IDs to derive a random matrix, and the sensory data can be recovered according to the received data vectors and the random matrix. The similar idea is adopted in [25] except that original data is recovered by linear programming. The authors in [26] extended the idea by proposing their own routing scheme. They also analyzed the capacity and delay for both single-sink and multi-sink networks under the proposed routing scheme.

Another related work for vehicle networks is proposed in [27]. It is assumed that the sampled data are *k*-sparse, where *k* is obtained by analyzing historical data. According to *k*, the number of the seed vehicles, *m*, is calculated centrally, where *m* determines the amount of compressed data that the sink will receive. During in-network transmission, the routing tree having *m* seed vehicles as leaf nodes is used. First, a new data collection process is initiated by each seed vehicle, and the distributed compressed sensing based algorithm, such as that shown in [24], is adopted to deal with such a process. After that, the sink will receive *m* compressed data.

The work in [28] proposed a new compressed sensing based data collection algorithm in order to detect outlier readings and broken links. Since the sparse property of outlier readings is different from that of the normal ones, different identity matrices for compression are used to discover outliers and broken links. However, the work neither provides any theoretical bound to guarantee detection accuracy nor clearly describes the compression rate.

Most of the existing compressive sensing based algorithms assume that sensory data have a known constant sparsity, which is not practical in WSNs. Therefore, the authors in [29] proposed an adaptive compressed data collection scheme in WSNs to deal with the situation in which the sparsity of sensory data varies with time and space. However, the computation at each node is complicated, which is also a challenge for sensor nodes since their computation ability and energy are limited.

Although the above algorithms save much energy since the compression bases are generated in a distributed manner and need not be broadcasted in a network, they all have the following problems. First, they do not consider the spatial-correlation so that the compression rate is not small enough by using a random matrix. Second, they assume that sensory data can be transformed into a sparse data matrix, which may not be practical for WSNs. Even if this is possible, its sparsity is hard to be obtained by the sink especially for large-scale WSNs. Third, since the random vectors are generated individually by the nodes, they cannot be orthogonal with each other, resulting in redundant information in the compressed data. Fourth, the information loss rate cannot meet an arbitrary user-specified precision requirement, so the dimension of the random matrix cannot be automatically adjusted accordingly.

Another compressive sensing based approximate data collection algorithm was proposed by [30]. The algorithm firstly decides the number of the required measurements, denoted by *M*, according to the sparsity of the sensory data using the compressive sensing technique. Then, *M* random walks are initiated to route and aggregate the required measurements to the sink node for further recovery. Such work was also based on the assumption that the sparsity of the sensory data is known. Meanwhile, the redundant information is also included in the compressed data since the random walk process may involve the same sensor many times.

In [31], an implementation method of the compressive sensing was provided on condition that the lossy nature of the WSN is sufficiently explored. The authors of [31] thought that not only the information of sparsity but also the spatial correlation among sensors were known ahead, and both of such prior pieces of information are helpful with compressing sensory data during collection. By combining both the random sampling matrix and the random selection matrix, no computation on sensory nodes was required while achieving the desired data reduction. Such a method saves lots of energy for each sensory node while implementing the compressive sensing techniques. However, the assumption of such seems a little strong.

The work in [32] proposed a hierarchy routing strategy to transmit and aggregate the data measurements returned by a compressive sensing technique. Based on [32], the whole network is divided into clusters, and the number of the required measurements in each cluster is decided according to the cluster size and the sparsity of the network. Since it still assumes that the sparsity of the whole network is known, the application of such schema is limited due to the strong assumption.

### 3.3. Hybrid Algorithms

Some researchers proposed combining the compressive sensing based algorithms with other ones, such as the straightforward packet forward method without compression.

The works in [33,34,35] proposed three hybrid algorithms in WSNs. In [33], the sensor nodes in the form of a spanning tree are grouped into two subsets, and only the ones that receive enough data apply a compressive sensing based method to compress data before transmitting them along the spanning tree towards the sink. In [34], the nodes are clustered based on the assumption that the distribution of nodes follows the Poisson point process. A compressive sensing based technique is applied at cluster-heads during the transmission. In [35], a packet forward strategy without compression is adopted during data collection.

The aforementioned algorithms can reduce energy consumption to some extent. However, they all have many drawbacks. For example, the algorithm in [33] requires that each node needs to hold the random vectors for other nodes, resulting in storage waste. The algorithm in [34] consumes lots of energy to cluster the nodes and broadcast the locations of clusters. The algorithm in [35] requires each parent node to wait for all of its children’s nodes’ data before transmitting, which increases the delay of data collection. Furthermore, the energy consumption could still be large since some sensory data are not compressed at the beginning and the additional information, such as sensor ID, still needs to be transmitted during data collection. Finally, these algorithms lead to imbalanced energy consumptions among the nodes in a network because the computation and transmission costs of some nodes are much larger than those of the other nodes.

The work in [36] studied how to combine the compressive sensing based technique with the principle component analysis technique, and proposed a novel data collection framework. It is assumed that sensory data can be converted to an *L*-sparse vector by the principle component analysis; thus, it only needs to sample data with probability L/N during data collection, where *N* is the size of the network and the compressive sensing based technique is used to forward data. Since the sparsity of sensory data varies, the authors also provided a method for adjusting the sampling probability as follows. First, since all sensory data from a network can be estimated by the compressed bases, the relative difference between the real collected values and their estimations can be calculated. If such a relative difference is larger than a given bound, the sampling probability is enlarged $C_{1}$ times; otherwise, it decreases $C_{2}/N$, where $C_{1}$ and $C_{2}$ are constants. The above data collection framework takes the advantages of both the compressive sensing based techniques and the principle component analysis techniques. However, it still has the following drawbacks. First, the randomly sampled data cannot guarantee to recover the original data even if the data are *L*-sparse. For example, if all the samples fall in one cluster, the data of the other clusters cannot be acquired and recovered. Second, the principle component analysis is based on historical data, which may be invalid at the current time when the sensory values vary frequently. Third, to estimate relative errors, the matrix used by the compressed sensing technique also needs to be transmitted in the network, resulting in more energy consumption. Fourth, the principle component analysis, the relative error computation and the sampling probability estimation are implemented centrally, which is not suitable for WSNs. Fifth, the sampling probability needs to be updated and broadcasted in every time slot, which increases energy consumption. Finally, the theoretical error bound of the above method is not provided, and the information loss rate cannot meet an arbitrary precision requirement.

In [37], the algorithms integrating compressive sensing and clustering were proposed based on block diagonal matrices. Firstly, the network is divided into clusters. Then, each cluster-head collected the sensory values within its cluster and generate Compressive Sensing (CS) measurements. Finally, such CS measurements were forwarded to the base station for recovery. Two routing strategies were also proposed in [37]. Such algorithms are efficient to reduce the scale of sensory data in a distributed manner. However, it still has the following problems. First, the block diagonal matrix may not be appropriate to describe the relationship among sensory data since the sensory values belonging to different clusters may be correlated with each other. Second, the authors decided the compression rate of each cluster based on the number of sensors in it; however, it may not be a good choice and the distribution of the sensory data should be also considered. Third, there also exists some redundant information since the correlations of the sensory values belonging in different clusters are not considered. Finally, the overhead of each cluster-head is quite high, so that it would lead to an imbalance of energy consumption in the whole network and shorten the lifetime of the WSN.

The JSM-2 model is introduced by [38] for data compression in sensor networks, where “*JSM*” is short for Joint Sparsity Model. According to the distributed compressive sensing theory, there are three different joint sparsity model (JSM), which are JSM-1, JSM-2 and JSM-3. The JSM-2 model is known as the Common Sparse Support model and requires that all the sensing signals are constructed from the same sparse set of basis vectors. Hence, the original sensory signals can be recovered using the same bases via Simultaneous Orthogonal Matching Pursuit algorithm. Due to the common sparsity structure of different sensing signals, the transmission cost can be largely reduced. However, the assumption in [38] also seems a little strong. Since the sensing data distribution of two sensor nodes may be quite different when the monitoring area is large, the common sparsity cannot be achieved and is impractical for this case.

According to the above discussion, most of these compressive sensing algorithms assume that the sparsity of the sensory data is constant and known. In practice, such an assumption seems too ideal since the sensory values continuously vary. Due to such a reason, the work in [39] proposed an adaptive data gathering schema which combined compressive techniques and network coding methods. Such schema allow the sink to query those interesting nodes adaptively for acquiring an appropriate number of measurements. Finally, in order to minimize the overall transmission cost, the authors also defined an NP-complete problem and proposed a greedy approximation algorithm to solve it. Such work is flexible and efficient for data collection; however, it only considers the scalar data and is not suitable to process multi-modal sensory data.

### 3.4. Summary

In conclusion, the idea of compressive sensing based data collection algorithms is to decrease the dimension of sensory data matrix as much as possible according to the users’ requirements. These methods are quite efficient and effective when the sensory data matrixes are sparse in certain subspaces. However, such a fact may not be always true and seems a little strict for WSNs and IoT systems. Even if the above fact is true, it is also quite hard to get the sparsity (i.e., which subspace makes sensory data to be sparse) of sensory data, so that the compression rate of such algorithms cannot be derived theoretically. Second, all of the algorithms have a fixed global information loss rate, which cannot meet an arbitrary user-specified precision requirement. Moreover, for the centralized algorithms of generating compression bases, the communication cost is huge since these bases need to be broadcasted in a network. For the distributed algorithms of generating compression bases, they cannot guarantee that the bases are orthogonal with each other since each basis is generated independently, so that there still exist some redundant information after compression and the compression rate cannot reach the optimal one either. The hybrid algorithms provide a valuable idea for approximate data collection. However, the existing works seem a little simple and have their own drawbacks.

## 4. Query-Driven Approximate Data Collection Algorithms

As important providers of sensory data sources to provide the sensory data, the WSNs and IoT systems also can be seemed as a database system, so that it is quite important for them to deal with various queries. Therefore, a group of data collection algorithms are designed accordingly. Since such data collection algorithms serve a certain query, the comment steps are as follows.

Firstly, based on the inputted query and the precision requirements, a distributed query plan, which involved a number of sub queries, is determined and diffused in-network.

Secondly, the proper sensory data set with a minimal size are retrieved by each sensor when it has received the corresponding sub query. Such partial results are transmitted and aggregated in the network toward the sink.

The main challenges of the algorithms in this category are how to determine the proper sensory data set for each query and how to aggregate the partial results, and the main advantage of these algorithms is that the transmission cost is extremely small since they are designed with the purpose of serving specific queries.

The existing works belonging to this category can be classified into two types, the sampling-based algorithms and the coding-based algorithms. The details are as follows.

### 4.1. Sampling-Based Algorithms

In many WSN applications, the statistic queries, such as aggregation and top-*k* queries, are the most popular ones. To answer such queries, the sampling techniques are good choices for designing an approximate data collection algorithm since only a small subset of sensory data needs to be transmitted.

The work in [40] proposed a sampling based approach to process top-*k* queries. The nodes with larger historical sensed values are selected to transmit data to the sink with a high probability. A group of linear programming functions are adopted to assign a sampling probability to each node. The algorithm is only applicable to top-*k* queries and is hard to be extended to other queries. Meanwhile, abnormal events are hard to be detected based on historical data.

The works in [41] proposed a uniform sampling algorithm to collect sensory data in order to process aggregation queries in static WSNs. The works in [42,43] introduced several Bernoulli sampling based algorithms to deal with aggregations in dynamic WSNs. The authors proved that the result accuracies can satisfy an arbitrary user-specified precision requirement, and the sampling ratio is quite small, i.e., only a few nodes are involved in data collection and the total communication cost is extremely small. However, similar to [40], these algorithms are only applicable to aggregations.

The works in [44,45] studied the sampling-based quantile computation algorithms. Although the algorithm in [44] has a fixed error bound and the work in [45] only considers one-hop networks, resulting in a large amount of energy saving, the collected data can only be used to obtain the quantile information. To overcome the above problem, the algorithms to retrieve *ϵ*-quantile and (ϵ,δ)-quantile were proposed by [46], using special data structure, tree-based routing and sampling techniques, the authors proved that the error bounds of the algorithms can be arbitrarily small. However, the algorithms in [46] only store the quantile information in-network and do not conserve the kernel information of the original sensory dataset.

Based on Bernoulli and uniform sampling techniques, another frequency and holistic aggregation algorithms were proposed by [47,48,49], respectively. In these works, the optimal sampling probability and sampling size are determined theoretically, so that the algorithms transmit the minimal size of sensory data during data collection while the desired query accuracies are guaranteed. Such algorithms save lots of energy during data collection; however, similar to the aforementioned ones, they are serving for specific queries and are not suitable for recovering original sensory datasets.

An energy-efficient data collection algorithm is proposed by [50] to process skyline queries for massively multidimensional sensory data, and several effective data reduction techniques were also introduced, such as dynamic filter, tuple-cutting strategy.etc. Such algorithm is also only suitable for dealing with skyline queries and not applicable for recovering original sensory datasets.

### 4.2. Coding-Based Algorithms

The basic idea of coding-based algorithms is to use coding techniques to reduce raw sensory data while guaranteeing accuracy. Some representative works are presented as follows.

A Bloom filter based approximate data collection protocol was studied in [51] in mobile WSNs. The nodes are partitioned into two categories, with collectors being responsible for collecting data and transmitting data to the sink, and non-collectors being responsible for sending data to reachable collectors. A collector first maps multi-dimensional values to real numbers and compresses them by the Bloom filter technique if its local buffer is full. Then, the collector transmits the stored values to the sink when the mobile sink is in its transmission range. According to the protocol, the original sensory values are hard to be recovered and the computation cost of collectors is high.

The work in [52] proposed a sketch based approximate data collection algorithms in WSNs. The algorithm requires each sensory value to be mapped to a binary sketch in order to compress data during transmission. However, this algorithm suffers from the same problem as [51] where it is hard for the compressed data to be recovered.

A joint coding-based data compression framework was proposed by [53] to reduce data redundancy during collection in in Wireless Multimedia Sensor Networks. The framework firstly uses an entropy-based divergence method to predict the compression efficiency of adopting the joint coding on the images collected by spatial-correlated cameras. A distributed multi-cluster coding protocol is then provided based on the prediction results. Although the framework guarantees that the overall compression rate of multimedia sensory data is maximized on the condition that the compressed data can be decoded by the sink with high precision, and the algorithm also produces redundant codings during data compression.

The work in [54] provided a compressive data collection strategy based on a novel data structure named *compression tree*. However, the problem of finding the optimal compression tree is NP-hard; thus, the complexities of the two algorithms in [54] are high. Furthermore, the ratio bound is not acceptable in real applications.

### 4.3. Summary

The query-driven approximate data collection algorithms are sufficient considering the users’ requirements during data collection, and they only transmit the data that are valuable to users so that many transmission costs are saved. However, these methods are only designed for specific kinds of queries. These algorithms do not consider correlations among sensory data and cannot recover original data. Furthermore, the coding-based algorithms still require all of the nodes to transmit their data to the sink, incurring a large communication cost. For the sampling-based algorithms, the core information of the raw data may not be preserved.

## 5. Other Works

For the convenience of reading the main advantages and disadvantages of the approximate data collection algorithms belonging to the three aforementioned categories are summarized in Table 1. Besides the aforementioned algorithms, there are some other approximate data collection works in WSNs.

The work in [55] discussed how to adjust the compression rate adaptively during data collection. Since a large compression rate can reduce communication cost but increase computation cost, it is important to find a trade-off. The authors of [55] proposed an algorithm for this problem. It is assumed that the data entropy of every node is available, and the lower bound $L_{i}$ of compression rate for node *i* satisfies $L_{i}=\frac{H_{i}}{i}$, where $H_{i}$ is the data entropy of node *i*. It is also assumed that $L_{i}\geq L_{j}$ if node *j* is on the path from *i* to the sink. Based on such assumptions, each node *i* computes the energy costs for communication and computation, respectively, during transmission. If the computation cost is larger, then the compression rate of node *i* is reduced to $L_{i}$. Otherwise, it keeps the same compression rate as its child. The problems of the above algorithm are as follows. First, the assumptions are too strict for WSNs, e.g., the data entropy of an internal node in a spanning tree not only depends on its own sensory values, but also depends on the data received from its child nodes; thus, it is hard to obtain the data entropy in advance. Meanwhile, the assumption on the lower bound of compression rate seems unconvincing since there does not exist any theoretical analysis to guarantee its correctness. Third, the algorithm only considers the compression problem on one path, which is not practical for WSNs since a network is usually organized as a tree. Finally, the algorithm cannot recover data with the information loss rate satisfying an arbitrary user-specified precision requirement.

To overcome the problem that the global information loss rate cannot be guaranteed, the authors in [56,57] proposed distributed algorithms to draw the dominant data set from big sensory data. The spatial correlations among different nodes are described by a correlation coefficient matrix. According to this matrix, the redundancy among data is reduced and the dominant data set can be extracted during transmission. For any given ϵ>0, it is proved that the proposed algorithm can return the dominant data set whose information loss rate is no larger than *ϵ*. Meanwhile, the distributed dominant data set drawing algorithm and the correlation coefficient maintaining algorithm have constant communication and computation complexities (O(1)) for each node. However, this algorithm also has several problems. First, it only reduces the amount of data in the spatial domain and does not consider the temporal domain. Furthermore, it is assumed that the sink has unlimited storage and processing ability, which may not be possible in some applications such as the ones employing smart phones or other hand-held devices for data collection.

## 6. Further Works

As shown in our Introduction section, approximate data collection is one of the most important issues for WSNs, and many efficient sensory data collection methods have proposed in recent years. However, with the appearance of IoT systems, there still exist some unsolved problems that need to be investigated in the future.

Firstly, the appearance of IoT systems make the amount of sensory data manifest explosive growth. As shown in Section 1, the number of connected “things” will reach 20.8 billion by 2020, and such a huge number of the intelligent “things” would generate a great number of sensory values, which make the amount of sensory data to exceed TB, PB even EB easily. In a word, the era of big sensory data is coming. The big sensory data bring us much information from monitoring physical world; however, the huge amount is also largely beyond the processing ability of most existing data collection techniques. Therefore, a series of new algorithms are eagerly required to collect big sensory data efficiently. Considering the existing techniques, the sampling is one of the feasible ways to design the lightweight and effective algorithms for dealing with big sensory data. However, most of the existing sampling based methods are designed for specific kinds of simple queries and do not support data recovery accurately. Thus, a group of new sampling based data collection algorithms are still worth being studied in the future.

Secondly, different from the traditional WSN, an IoT system has various types of sensor nodes. For example, an intelligent traffic monitoring system could include many monitoring sensors, e.g., electronic eyes, GPS devices, intelligent traffic lights, etc. Such sensors will sample multiple types of sensory data from the monitoring environment. A crowd sourcing application based on smart phones might utilize the sensors embodied in it, including accelerometer, digital compass, gyroscope, GPS, microphone, camera and so on. Therefore, sensory data sampled by these sensor nodes also consist of various modality, that is, the scalar data, the vector data, the multimedia data, etc. are always involved in the same sensory dataset, and the variety of sensory data bring many challenges for data collection. However, the current works on sensory data collection only focus on single modular data and rarely consider the problem of how to collect multi-modal sensory data efficiently. To solve the above problem and make multi-modal sensory data be collected cooperatively, the correlation among different types of sensory data should be analyzed thoroughly. Then, new frameworks, which can make multiple types of sensory data become integrated, are going to be constructed. Finally, new distributed and energy-efficient data collection algorithms need to be designed according the above correlations and frameworks. In order to achieve the three steps above, the stochastic process based methods and the time series analysis, etc. are suitable to be adopted.

Thirdly, although the amount of raw big sensory data sets is quite huge, there exists massive redundant information in them since the sensory data are strongly correlated with each other. Considering that the network’s energy and resource are quite limited for WSNs and IoT systems, we only want to transmit and collect the useful sensory dataset instead of the raw ones. We call such datasets kernel datasets, and the method of retrieving the kernel dataset from big sensory data is rarely taken into account by the existing works, although it is significant for dealing with big sensory data. Therefore, a series of new techniques to draw kernel datasets in WSNs and IoT systems need to be explored in the future. To achieve such an aim, the *global information loss rate*, which is an important metric to evaluate the quality of kernel dataset, should be firstly defined. To the best of our knowledge, the work in [56] is the unique literature to give the formal definition of the *global information loss rate* based on statistics. However, the metric in [56] is suitable for the scalar data; moreover, it also requires that the sensors in the network should be synchronous and sample sensory data with the same frequency. Such constraints may be common for WSNs, but seem a little strong for IoT systems, especially when multi-modal sensory data are involved. Thus, the complicated correlations among sensory data should be taken into account, and the new metrics to evaluate the global information loss rate need to be reconsidered. Since the sensory data can be regarded as the information carriers, the information entropy based metric may be a good choice to define the *global information loss rate*, and the entropy based techniques may be valuable for designing the kernel dataset drawing algorithms.

Fourth, the sensor nodes in IoT systems are usually very cheap and their abilities on sensing, computation, storage and communication are quite limited, so that the quality of sensory data is extremely low. Furthermore, some of the battery-free nodes may also be involved in some IoT systems, which makes the quality of sensory data to be even harder for controlling. Taken the currency as an example, the battery-free sensor nodes in a given IoT system cannot sensory data if the energy gained from environment does not exceed a certain threshold, and thus it cannot guarantee that the object is monitored in real time. Similarly, the precision, completeness and consistency of a sensory dataset also cannot be guaranteed in some IoT systems. Thus, the sensory data should be repaired during collection in order to provide the high quality dataset to users. Unfortunately, few of the existing methods have considered such a problem. Therefore, the cooperative data repairing techniques during collection are also worth being studied in the future. Similar to constructing the kernel data drawing methods, the correlations among the sensory data are very useful for designing cooperative data repairing algorithms, where the logistics based techniques, stochastic process based models (e.g., continuous Markov model) *etc.*, can be utilized for sensory data repairing.

Finally, the data collections to support the advance applications deserve to be studied intensively in the future as well. For example, visualization has become a popular application for networks and provides a friendly interface for users to observe and comprehend the physical worlds. However, almost none of the existing data collection methods consider how to support such an application. Furthermore, in future IoT systems, the data collectors may no longer be computers, and they could be smart phones, Pads or other small hand-held devices, so that the data collectors may have limited abilities on processing and exhibition. These limitations should be sufficiently taken into consideration in order to transmit the processable sensory data only and save energy during data collection.

## 7. Conclusions

Since data collection is a fundamental topic for both WSNs and IoT systems, lots of efficient and effective data collection algorithms have been proposed. This paper summarizes the existing contributions on sensory data collection into four categories, introduce their main ideas, analyzed their advantages and pointed out their disadvantages. Finally, we are looking forward to the future research problems on such topics and provide five unsolved ones that are quite worthy of being carefully studied.

## Figures and Tables

**Table 1:** Comparisons among different approximate data collection algorithms.

	Properties	Advantages	Disadvantages
Algorithms			
Model Based Algorithms		(1) The spatial and temporal correlations among sensory data are considered (2) Only a partial of sensory values need to be transmitted	(1) The mathematical model are usually too ideal (2) Lots of in-network communication are involved to determine parameters of prediction models and guarantee the consistence between local and global models (3) The algorithm has the fixed error bound and cannot be adjusted automatically
Compressed-Sensing Based Algorithms		(1) The compress ratio can be controlled according to users’ requirements (2) Efficient when the sensory data matrix are sparse	(1) The assumption is strong and the sparsity of sensory data is very hard to be obtained (2) All algorithms have the fixed global information loss rate (3) The centralized algorithms cost too much to transmit the bases, while the distributed one also have redundant information since the bases are not orthogonal
Query-Driven Algorithms		(1) The transmission cost is extremely small (2) Sufficiently consider the users’ requirement and only transmit the valuable sensory data	(1) The algorithms are only designed for the specific kind of queries (2) They do not consider the correlations among the sensory data and cannot support to recover the original sensory data (3) The kernel information of the sensory data is not preserved
